# 

**Published:** 1861-01

**Authors:** 


### TO CORRESPONDENTS.

By a recent change in the management of the Journal, its whole business affairs will hereafter come under the supervision of the Editor, and it is therefore requested that ALL Communications, whether relating to the Editorial, Subscription, Advertising or Financial Departments, should be addressed to J. G. WESTMORELAND, or Editor of Medical and Surgical Journal, Atlanta, Ga.

